# A novel sensor-based assessment of lower limb spasticity in children with cerebral palsy

**DOI:** 10.1186/s12984-018-0388-5

**Published:** 2018-06-04

**Authors:** Seoyoung Choi, Yong Beom Shin, Soo-Yeon Kim, Jonghyun Kim

**Affiliations:** 1 Department of Robotics Engineering, DGIST (Daegu Gyeongbuk Institute of Science and Technology), 333 Techno Jungang-daero, Daegu, 42988 Republic of Korea; 2 Department of Rehabilitation Medicine, Pusan National University School of Medicine and Biomedical Research Institute, Pusan National University Hospital, 179 Gudeok-ro, Busan, 49241 Republic of Korea; 3 Department of Rehabilitation Medicine, Pusan National University Yangsan Hospital, 20 Geumo-ro, Yangsan, 50612 Republic of Korea

**Keywords:** Accuracy, Assessment, Cerebral palsy, Inertia measurement unit (IMU), Joint angle, Modified Tardieu scale, Reliability, Spasticity

## Abstract

**Background:**

To provide effective interventions for spasticity, accurate and reliable spasticity assessment is essential. For the assessment, the Modified Tardieu Scale (MTS) has been widely used owing to its simplicity and convenience. However, it has poor or moderate accuracy and reliability.

**Methods:**

We proposed a novel inertial measurement unit (IMU)-based MTS assessment system to improve the accuracy and reliability of the MTS itself. The proposed system consists of a joint angle calculation algorithm, a function to detect abnormal muscle reaction (a catch and clonus), and a visual biofeedback mechanism. Through spastic knee and ankle joint assessment, the proposed IMU-based MTS assessment system was compared with the conventional MTS assessment system in 28 children with cerebral palsy by two raters.

**Results:**

The results showed that the proposed system has good accuracy (root mean square error < 3.2°) and test-retest and inter-rater reliabilities (ICC > 0.8), while the conventional MTS system has poor or moderate reliability. Moreover, we found that the deteriorated reliability of the conventional MTS system comes from its goniometric measurement as well as from irregular passive stretch velocity.

**Conclusions:**

The proposed system, which is clinically relevant, can significantly improve the accuracy and reliability of the MTS in lower limbs for children with cerebral palsy.

## Background

Cerebral palsy (CP) is defined as a non-progressive brain disorder of movement and posture. Most children with CP experience spasticity, a motor disorder caused by increased tonic stretch reflexes [1], due to upper motor neuron syndrome [2]. CP children have difficulties walking independently due to abnormal posture and gait, and they have joint deformity and pain in severe cases. In particular, lower limb spasticity mostly accompanies clonus, an involuntary, rhythmic, muscular contraction and relaxation [2, 3].

Spasticity assessment has been used to predict the severity of CP in activities in daily life (ADL) [1, 4]. It is also an important tool in determining the effect of interventions, including rehabilitation programs [2], botulinum toxin injections [2, 3], and orthopedic surgeries [1]. Moreover, the level of clonus needs to be assessed because clonus can cause instabilities during joint motions or weight bearings [2, 4, 5]. However, the accuracy and reliability of the clinical assessments of spasticity are quite low owing to the subjectivity of the rater [6]. Of those, the Modified Ashworth Scale (MAS) is the most widely used because of its convenience; it is performed with passive stretching by the rater, without any special tool. Since the MAS majorly depends on the characteristics of the resistance felt during the manual passive stretch, the rater highly relies on the subjective feeling, which is sensitive to the passive stretch velocity (PSV) owing to the velocity dependency of spasticity [7, 8], and thus has a low reliability. In addition, the MAS has a fundamental limitation in that clonus cannot be assessed [6]. As a solution to these problems, the Modified Tardieu Scale (MTS) [9], which is described in detail in the method section, was proposed to reduce the rater’s subjectivity by utilizing a goniometer to consider the velocity dependence, and this provide a guideline for two different PSVs (slow and fast) to assess clonus [10, 11]. Nevertheless, the accuracy of the MTS is still poor and its reliability has been questioned due to the inaccuracy of the goniometric measurement and the ambiguous (subjective) description of PSV in the guideline, especially for the “as fast as possible velocity” [2, 4, 6, 11].

There were some attempts to improve the spasticity (and/or clonus) assessment. Several custom devices with multiple sensors were developed for more objective assessments [12–14]. Since, instead of the MAS or MTS, less verified parameters which were measured by using the devices were proposed for spasticity assessment, it was difficult to use them immediately in the clinical setting. Few attempts utilized robots or robotic devices to improve the accuracy/reliability of the MAS or MTS [13–16]. However, these were also inadequate to be applied in the clinical setting, owing to their expensive and complex systems [17]. Moreover, all studies did not clearly show whether they can be used for clonus assessment [12, 14, 15].

Recently, several studies have investigated the MTS by using the inertial measurement unit (IMU) to improve the accuracy/reliability problems mentioned above because an IMU sensor has relatively low cost and is easy to use [18, 19]. For the MTS assessment, both joint angle measurement and muscle reaction (catch or clonus) [2] detection using IMU are essential. Since most studies used a magnetometer in IMU for an accurate joint angle measurement, they were not appropriate in the clinical settings where the heterogeneity of the earth’s magnetic fields becomes significant due to ferromagnetic and other magnetic materials used in medical devices [19, 20]; to overcome the heterogeneity requires inconvenient calibration process of the sensor with special setup [21–23]. A study used a gyroscope without magnetometer, but showed a poor accuracy of joint angle measurement [3]. Moreover, all existing studies on IMU-based MTS assessment lacked efforts to provide regular PSVs for reliable muscle reaction detection [21]; pendulum test [22, 24] and a use of metronome [23] were reported to induce raters to control the PSV, but resulted in inaccuracies as well as inconveniences [22]. In addition, all existing IMU-based MTS assessments have a limitation in that clonus assessment was not considered [3, 21, 22].

This study proposed a clinically relevant IMU-based MTS assessment system to improve the accuracy and reliability of lower limb (knee and ankle) spasticity evaluation in children with CP. For clinical use, a joint angle calculation algorithm using IMU was developed without magnetometer. An acceleration mapping scheme using rotation matrix was included in the algorithm for better accuracy. We also developed a method for detecting muscle reaction considering clonus (i.e., duration of clonus). Moreover, for better reliability of muscle reaction detection, a visual biofeedback based on IMU was developed in the graphic user interface (GUI) of the proposed system in order to provide regular PSVs [25]. In addition, we added an IMU attachment guideline for each target joint to the GUI to maintain the convenience of the existing clinical assessments. For validating the IMU-based MTS assessment using the proposed system (iMTS), five normal subjects were tested to confirm the accuracy of the joint angle calculation method using IMU, and a clinical trial of 28 patients with CP (18 knee and 10 ankle joints) was conducted to evaluate the test-retest and inter-rater reliabilities of the iMTS by comparing it with the conventional MTS assessment and to verify its viability in clinical practice. The originality of the proposed iMTS is summarized in Table 1 by comparing the existing IMU-based MTS (and spasticity) assessments.

**Table 1:** Comparison of MTS (spasticity) assessments using IMU

	Existing studies	Magnetometer	Calibration	Clonus	Regular PSV	Accuracy	Reliability	Target
MTS assessment using IMU	[21]	X	X	X	X	O	unknown	Knee joint (CP *n* = 20)
	[23]	X	X	X	△	X	O	Elbow joint (stroke *n* = 13)
	[3]	O	X	X	X	X	unknown	Ankle joint (CP *n* = 4)
Spasticity assessment using IMU	[22]	X	X	X	△	X	O	Knee joint (stroke *n* = 11)
	[12]	O	X	X	X	X	O	Knee / ankle joints (CP *n* = 28)
Proposed system	iMTS	O	O	O	O	O	O	Knee / ankle joints (CP n = 28)

The symbol in the Magnetometer column indicates whether it requires a magnetometer (X) or not (O); the symbol for Calibration indicates whether it requires calibration (X) or not (O); the symbol for Clonus indicates whether it considers clonus (X) or not (O); the symbol for Regular PSV indicate if it is achieved (O), incompletely achieved (△), and not considered (X); the symbol for Accuracy indicates whether the root mean square error of the joint angle is less than 4 deg. (X) or not (O); and the symbol for Reliability denotes whether both test-retest and inter-rater reliabilities are consistent (ICC > 0.8) (X) or not (O)

## Methods

### Required functions and characteristics of target joints

**Fig. 1:**
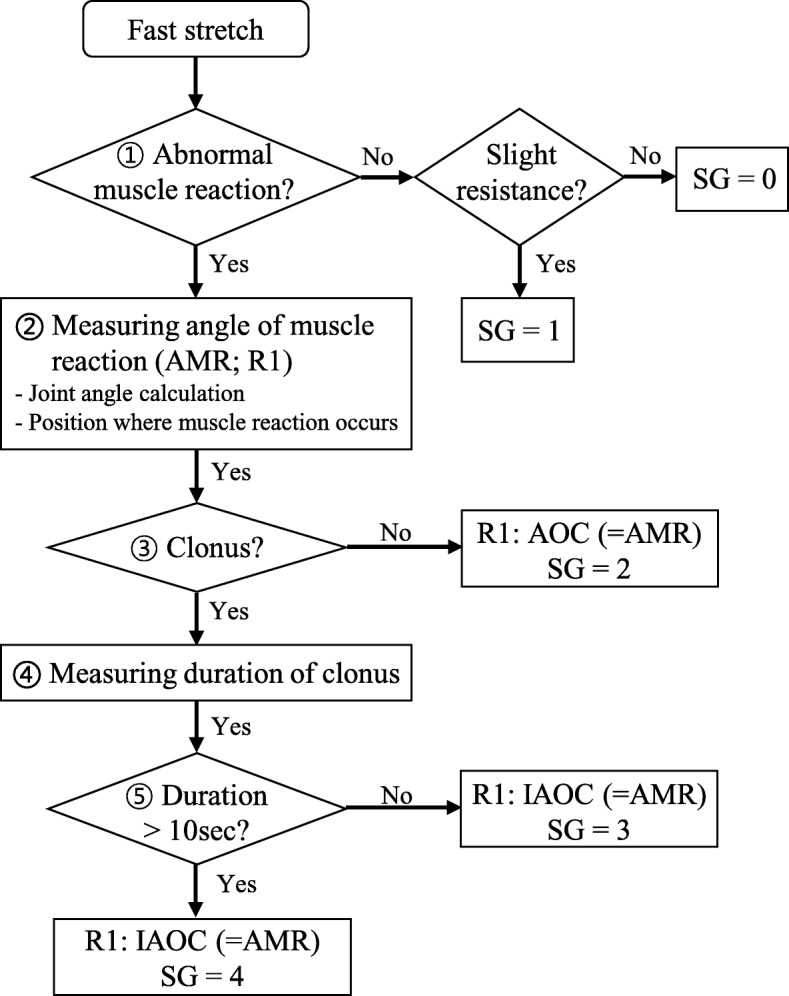
Schematic diagram of the MTS assessment. Except ROM measurement. SG: spasticity grading; AMR: angle of muscle reaction; AOC: angle of catch; IAOC: initial angle of clonus

The MTS assessment procedure [6], except the step measuring range of motion (ROM; called R2 in MTS) is illustrated in Fig. 1. Despite fast stretching, the clinician can detect abnormal muscle reaction, including catch and clonus (#1 and #3 in Fig. 1) [10, 11]. However, it is difficult to accurately measure the angle of muscle reaction (AMR; called R1 in MTS). Hence, iMTS requires 1) calculation of the joint angle, 2) detection of the location of occurrence of muscle reaction, and 3) measurement of the duration of clonus (#4 and #5 in Fig. 1). In addition, it is important 4) to provide regular PSVs during fast stretching [25] due to the velocity dependency of spasticity [6].

The target joints in this study, namely, the knee and ankle, mainly exhibit flexion/extension and dorsiflexion/plantarflexion, respectively, in a two-dimensional sagittal plane [26]. However, non-sagittal movements are possible, and some of these movements such as internal/external rotation of the knee and inversion/eversion of the ankle, are not negligible at near full flexion/extension and dorsiflexion/plantarflexion [26] due to their anatomical chracteristics [27]. Considering its clinical importance, this study focused on the spasticity assessments of three major muscles: the knee flexor and extensor, and ankle plantar-flexor [2]. Each initial posture and passive stretch required for MTS according to the MTS instructions are summarized in Table 2.

**Table 2:** Instruction of MTS assessment

Joint	Knee joint		Ankle joint
	Extensor (Quadriceps)	Flexor (Hamstrings)	Plantar flexor (Calf muscle)
Hip	90^°^ flexion		neutral position
Knee	maximum extension to maximum flexion	maximum flexion to maximum extension	full extension
Ankle	N/A		maximum plantarflexion to maximum dorsiflexion

Note that MTS assessment is conducted in supine position

### Proposed IMU-based MTS assessment system

#### Joint angle calculation

**Fig. 2:**
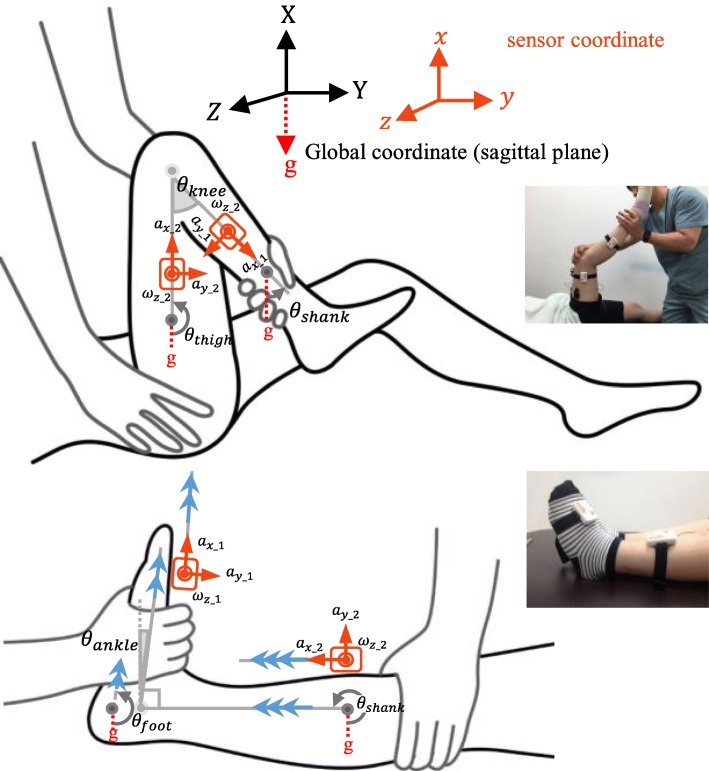
Definition of segment/joint angles and coordinates

To calculate the joint angle, each segment angle should be obtained, and then the relative angle should be calculated. According to the definition of each angle shown in Fig. 2, each joint angle can be obtained as follows:

1 $$ \left\{\begin{array}{c}{\theta}_{knee}={\theta}_{shank}-{\theta}_{thigh}+{180}^{\circ },\\ {}{\theta}_{ankle}={\theta}_{foot}-{\theta}_{shank}+{90}^{\circ },\end{array}\right) $$

where *θ*_*thigh*_, *θ*_*shank*_, and *θ*_*foot*_ denote the segment angles. IMUs were attached to obtain the segment angles in (1), and the attachment locations and orientations of the IMUs are shown in Table 3 and Fig. 2. The locations were chosen to ensure stable attachment by minimizing skin artifacts [28]. Note that inertial effects, skin deformation, and sliding near joints as well as skin deformation due to muscle contraction caused the skin artifacts [29, 30]. In addition, it should be noted that there are two IMU locations chosen for shank (Table 3 and Fig. 2); the chosen location for the ankle joint cannot be used for the knee joint because of noticeable skin artifacts (inertial effects and sliding) caused by significant movement of the shank (manipulated segment in the knee joint); further, the location for the knee joint is also not appropriate for the ankle joint because of the skin deformation due to the contraction of the ankle plantar-flexor [29, 31]. The orientations of the IMU were determined to be as close as possible to the sagittal plane (global XY plane in Fig. 2).

**Table 3:** Anatomical landmarks for attaching the IMU

	Knee joint	Ankle joint
Manipulated segment	Shank: the lateral 1/3 surface of the shank, from the malleolus	Foot: the anterior 2nd metatarsal bone
Held segment	Thigh: the lateral 1/3 surface of the thigh, from epicondyle of the femur	Shank: the anterior 1/3 surface of the shank, from the tibia

The sensor coordinates of the attached IMUs (x-y-z axes) are shown in Fig. 2. The method of determining the coordinates was as follows. Since the main movements of the target joints (knee flexion/extension and ankle dorsiflexion) appear on the sagittal plane, the normal direction of the plane was set to the z-axes of two attached IMUs [26]. Each x-axis of the IMU coincided with the rotational axis of each additional movement (shank/thigh internal/external rotation in knee flexion/extension and foot inversion in ankle dorsiflexion) that was not on the sagittal plane, as mentioned in the first subsection. Note that the additional movement of the thigh can be caused by the spasticity pattern of CP [1, 2].

For clinical use, we used only the accelerometer/gyroscope of the IMU without the magnetometer to obtain the segment angles. The accelerometer is appropriate for quasi-static states [32] but is inaccurate for dynamic states owing to the additional acceleration caused by its motion [33]. Conversely, the gyroscope that can be used in the dynamic state only obtains the relative angle by integrating the angular velocity measured [34]; thus, the segment angles (absolute angle) depend on the initial value. Moreover, it would have a drift error when used continuously [35]. In this study, we proposed a method to calculate the segment angles by selecting a suitable sensor for each state (quasi-static or dynamic) that is determined by the measured data from the IMU [25]. In the quasi-static state, the acceleration measured mainly comes from gravity, and the angular velocity measured was very small. Hence, we defined the quasi-static state as if the following conditions were met:

2 $$ \left\{\begin{array}{c}\mid g-\operatorname{}\sqrt{a_{x\_1}^2+{a}_{y\_1}^2+{a}_{z\_1}^2}\mid <{a}_{qs\_ th}\\ {}\left|{\omega}_{z\_1}\right|<{\omega}_{qs\_ th}\end{array}\operatorname{}\right) $$

where *a*_*i*_1_ (i = x, y, and z) and *ω*_*z*_1_ denote the i-axis linear acceleration and angular velocity measured by the IMU attached in the manipulated segment by the rater. If (2) was not met, it was regarded as the dynamic state. Note that the small thresholds *a*_*qs*_*th*_ and *ω*_*qs*_*th*_ were experimentally selected as 0.2 m/s^2^ and 10 deg./s, respectively.

Assuming that each segment movement occurs only in the sagittal plane, the accelerometer can be used as a tilt sensor in the quasi-static state (2) [36], and the segment angles in the dynamic state can also be obtained by integrating the angular velocity measured from the gyroscope as follows [35]:

3 $$ {\theta}_n=\mathrm{atan}2\left({a}_{y\_n},{a}_{x\_n}\right) $$

4 $$ {\theta}_n=\int {\omega}_{z\_n}(t) dt+{\theta}_{n\_ latest} $$

where *a*_*x*_*n*_ and *a*_*y*_*n*_ denote the x and the y-axis acceleration measured by the accelerometer of the IMU attached on the n segment (shank, thigh, and foot), respectively; *ω*_*z*_*n*_ the z-axis angular velocity measured by the gyroscope of the IMU; and *θ*_*n*_*latest*_ the latest angle obtained by (3) with the accelerometer. However, as mentioned in the first subsection, the significant non-sagittal plane movements coexist at near full flexion/extension [26]; thus, even if the IMUs were initially well attached to align the plane (*a*_*z*_*n*_ are near zero), they would be outside of the plane. Since the movements occur in the quasi-static state, (3) would result in a large segment angle error at near full ROM.

**Fig. 3:**
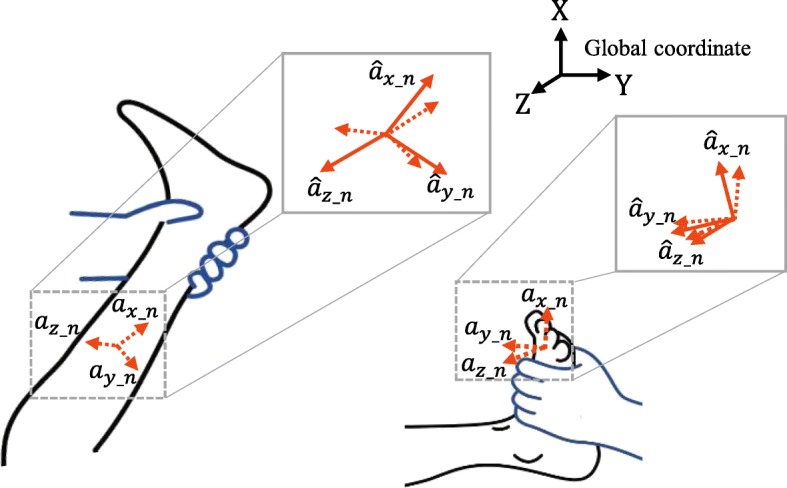
Compensation scheme of the x-axis rotation using rotation matrix

Hence, when the non-sagittal movements significantly occurred, we applied a mapping scheme based on a rotation matrix to obtain the net segment rotation on the sagittal plane (along the Z-axis in the global coordinate) only, as illustrated in Fig. 3. Since *a*_*z*_*n*_ (*n* = 1 or 2) becomes non-zero when each segment was outside of the sagittal plane due to those movements (Fig. 3), we used the following condition to determine whether the scheme was needed:

5 $$ {a}_{z\_n}>\left|{a}_{sp\_ th}\right| $$

where *a*_*qs*_*th*_, a small threshold, was heuristically selected as 2 m/s^2^. If (5) was satisfied, the measured IMU accelerations need to be transformed using the mapping scheme. From the definition of rotation matrix based on the z-y-x Euler angles (*α*, *β*, and *γ*) [37], if the rotation of the IMU along the x and y axes (*β* and *γ*) is compensated by multiplying the rotation matrix, the z-axis of the IMU coincides with the global Z-axis (Fig. 3) and thus (3) can be used to obtain the segment angles on the sagittal plane. Here, the mapping of the accelerations using the rotation matrix for the compensation was as follows:

6 $$ \left[\begin{array}{l}{\widehat{a}}_{x\_n}\\ {}{\widehat{a}}_{y\_n}\\ {}{\widehat{a}}_{z\_n}\end{array}\right]={\displaystyle \begin{array}{l}\mathrm{U}\\ {}\mathrm{S}\end{array}}\mathbf{R}\left[\begin{array}{l}{a}_{x\_n}\\ {}{a}_{y\_n}\\ {}{a}_{z\_n}\end{array}\right], $$

7 $$ \mathrm{with}\ {\displaystyle \begin{array}{l}\mathrm{U}\\ {}\mathrm{S}\end{array}}\mathbf{R}=\left[\begin{array}{ccc} c\alpha c\beta & c\alpha s\beta s\gamma - s\alpha c\gamma & c\alpha s\beta c\gamma + s\alpha s\gamma \\ {} s\alpha c\beta & s\alpha s\beta s\gamma & s\alpha s\beta \gamma - c\alpha s\gamma \\ {}- s\beta & c\beta s y& c\beta c\gamma \end{array}\right] $$

where script S and U denote the sensor coordinate and the coordinate in which the global coordinate is only rotated along the Z-axis, respectively. Note that cα is cos(α), and sα is sin(α). For (7), we used *α* = 0 not to compensate the segment rotation on the sagittal plane, and *β* and *γ* were obtained as follows [33]:

8 $$ \left\{\begin{array}{l}\beta =\mathrm{atan}\ 2\left({a}_{z\_n},{a}_{x\_n}\right);\\ {}\gamma =\mathrm{atan}2\left({a}_{z\_n},\sqrt{a_{x\_{n}^2}+{a}_{y\_{n}^2}}\right)\end{array}\right) $$

After the mapping, the segment angles were obtained from (3) using the compensated ${\widehat{a}}_{x\_n}$, ${\widehat{a}}_{y\_n}$, and ${\widehat{a}}_{z\_n}$.

**Fig. 4:**
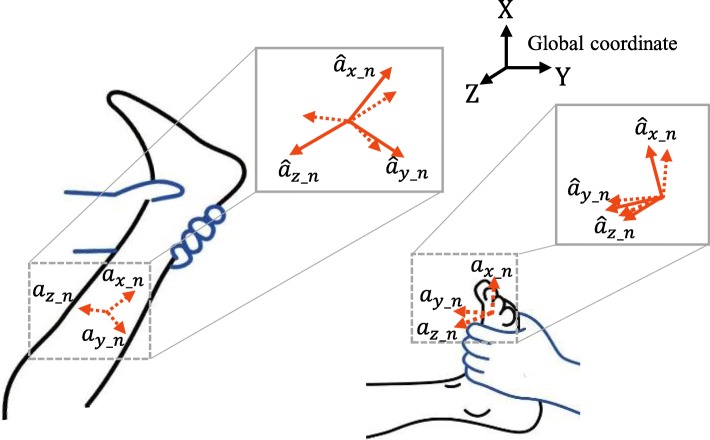
IMU-based joint angle calculation algorithm

In summary, we proposed a joint angle calculation method based on IMU as shown in Fig. 4. It is an effective measurement method for knee and ankle motions, especially with the non-sagittal plane movements and without a magnetometer. This method can also be used to obtain ROM (R2) in the iMTS. During a slow passive stretch to measure ROM, the MTS allows the raters to stop the stretch when they have reached the subjects’ ROM limit based on the subjects’ haptic feeling [38]. Hence, the maximum (knee extension and ankle dorsiflexion) or the minimum (knee flexion) joint angle calculated by the IMU during the slow stretch can be regarded as the ROM. Compared with goniometric measurements in the MTS, the proposed method can be more convenient to use alone and can increase measurement accuracy.

#### Muscle reaction (catch and clonus) detection

**Fig. 5:**
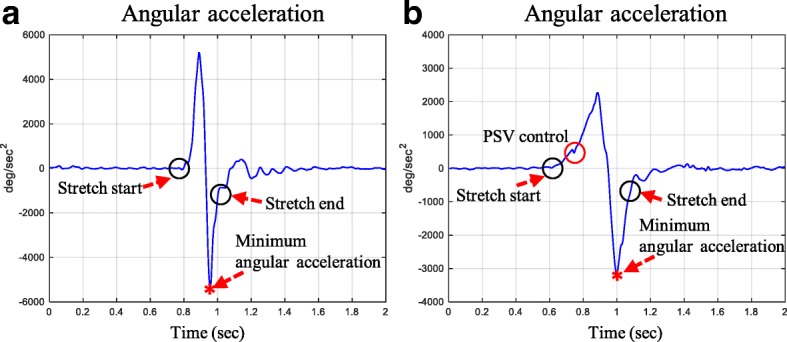
Typical angular acceleration profiles during fast stretching

**Fig. 6:**
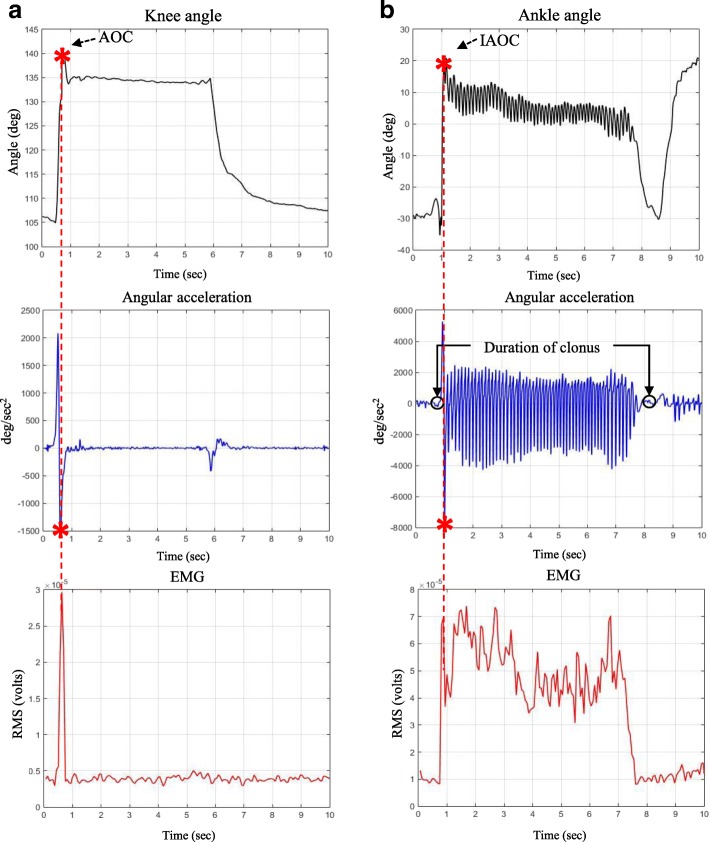
Typical knee/ankle joint angles, angular accelerations, and EMG data during fast stretching

In addition to the proposed joint angle calculation above, the muscle reaction should be detected to obtain the AMR (Fig. 1). For the MTS, the clinician rapidly accelerates to provide fast PSVs; thus, the joint angular acceleration monotonically increases before the muscle reaction. When muscle reaction occurs, the acceleration is suddenly and greatly decreased due to the reflex torque caused by the muscle reaction (Euler’s 2nd law) [39], as displayed in Fig. 5a. Of course, it is possible that the rater adjusts the angular acceleration before the muscle reaction only for PSV control, as shown in Fig. 5b. However, the acceleration decrease in this unusual case is negligible, compared with the decrease due to the muscle reaction (Fig. 5b). Therefore, we obtained the AMR as the position at which the angular acceleration was minimized between the start and the end of the stretch [39], as shown in Figs. 5 and 6. It allows the clinician to determine the AOC or IAOC according to the type of the muscle reaction (#3 in Fig. 1).

Furthermore, it is necessary to measure the duration of clonus (oscillatory movement) because clonus is divided into fatigable (< 10 s) and unfatigable (> 10 s) based on the duration (#5 in Fig. 1) [11]. The duration was obtained by measuring the time interval between the IAOC to the condition when the angular acceleration approaches zero, as displayed in Fig. 6b, and the condition was detected as follows:

9 $$ {\sigma}_w>3{\sigma}_{initial} $$

where *σ*_*w*_ denotes the standard deviation of ${\ddot{\theta}}_{knee}$ (or ${\ddot{\theta}}_{ankle}$) in 1-s time window and *σ*_*initial*_ the standard deviation of ${\ddot{\theta}}_{knee}$(or ${\ddot{\theta}}_{ankle}$) during the initial no movement condition.

Figure 6 shows two typical examples of AOC/IAOC and the duration of clonus obtained by the proposed method. The EMG supports the validity of the detected muscle reaction as well as the end of clonus.

#### Visual biofeedback

As mentioned in background chapter, providing regular PSVs is also important to improve the reliability of spasticity assessment. To achieve the regular PSV, pendulum test [22, 24] and use of metronome [23] were reported, but had many limitations to be used (Table 1). The pendulum test based on natural drop due to gravity requires a fixed initial posture, and causes insufficient PSV to induce spasticity [22]. The metronome only provides the time duration constantly, and thus it cannot restrict PSV directly [23].

**Fig. 7:**
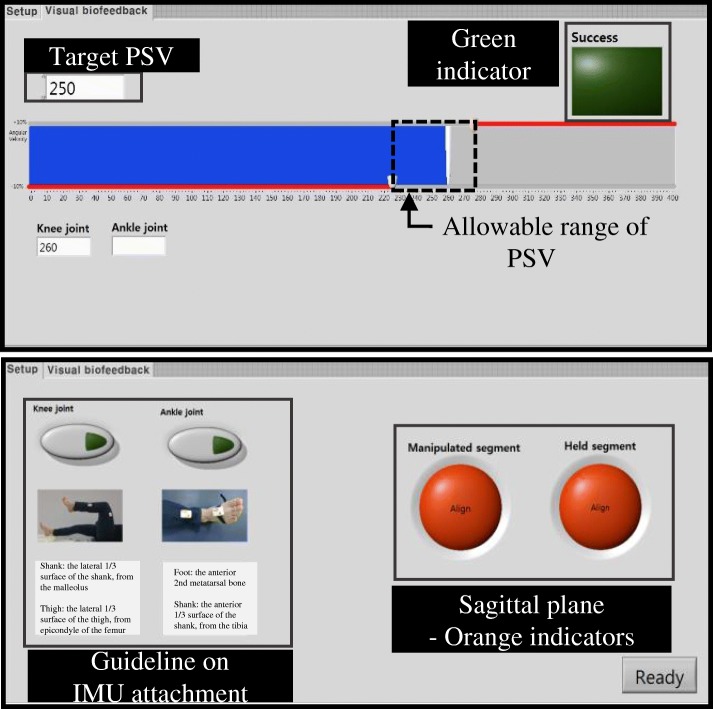
Graphic user interface of the proposed system

As such, we developed a visual biofeedback, which has recently been reported [25], and included it in a GUI for the proposed iMTS (Table 1), as shown in Fig. 7a. The visual biofeedback was to help the clinician regularly provide a target PSV, which was selected as a sufficiently fast velocity for a subject to evoke abnormal muscle reactions during passive stretching. Using GUI, it displayed both the allowable range of PSV (from 90 to 110% of the target PSV) and the PSV measured by the gyroscope of the IMU (*ω*_*z*_1_ + *ω*_*z*_2_), as a red solid line and blue bar, respectively (Fig. 7a) [26]. To check easily whether the PSV provided was well regulated, we added a green indicator that turns on when the maximum actual PSV is within the range (Fig. 7a).

It is noteworthy that determining the target PSV is essential for the visual biofeedback. However, it remains unclear how the PSV for the fast stretch should be selected [40]. Hence, in this study, the target PSV was determined as the average of the three maximum PSVs that were collected by the first clinician’s (rater’s) three valid fast stretches wherein the clinician performed the stretch within 1 s and felt an abnormal muscle reaction [10, 11, 40].

#### Implementation

To implement the proposed IMU-based MTS assessment system, two IMUs (shimmer3, the shimmer, Ireland) consisting of a three-axes accelerometer, gyroscope, and magnetometer were used (Table 3). Note that the magnetometer of the IMU was not applied in this study. The raw data (acceleration and angular velocity ω) of the IMUs were collected at a sampling rate of 204.8 Hz, and a second-order Butterworth low-pass filter with 10-Hz cutoff frequency was applied to remove noise from the raw data.

The raw data collected by the IMU were transmitted to the PC through a Bluetooth communication. As mentioned above, the visual biofeedback was implemented as a GUI by LABVIEW (National Instruments, Austin TX, USA). The other part of the GUI, which was used to help the clinician attach the IMUs at the initial stage of the iMTS, provided a guideline image on IMU attachment for each target joint and showed whether the attached IMUs were located on the sagittal plane (orange indicators), as displayed in Fig. 7b. The custom program installed in the PC was developed by MATLAB (MathWorks, Inc., Natick, MA, USA) to implement the joint angle calculation algorithm and muscle reaction detection method for measuring the ROM, AMR, and duration of clonus.

## Experiments

### Experimental setup

To evaluate the proposed iMTS, we designed two experiments: the accuracy study with healthy subjects and the reliability study with patients. In the accuracy study, only the accuracy of the joint angle obtained by the proposed IMU-based algorithm was verified by comparing it with the motion capture system (Mocap; Bonita, Vicon, UK) during the healthy subjects’ active stretch without abnormal muscle reaction. Note that the AMR and duration of clonus were not measured in the study. For the Mocap, markers were placed to the lower limbs based on the plug-in-gait model [37, 41], and the stretch motions were captured at a sampling rate of 250 Hz. The IMUs attached and the Mocap were synchronized using a DAQ board (National instruments, Austin TX, USA).

The reliability study with patients was conducted to compare the test-retest and inter-rater reliabilities of conventional MTS (cMTS) and iMTS. Two channels of EMG (Trigno wireless EMG, Delsys Inc. USA) were attached to the target muscles introduced in Table 2 to confirm the existence and the timing of the abnormal muscle reaction due to spasticity.

### Participants

Five healthy subjects (three males; age 26.0 ± 2.0 years; height167.2 ± 6.9 cm; weight 68.2 ± 11.3 kg) without surgery history and pain in the lower limbs participated in the accuracy study. They signed the informed consent approved by the Daegu Gyeongbuk Institute of Science and Technology institutional review board (IRB) prior to the experiment (No. DGIST-160114-HR-005-03).

As summarized in Tables 4, 28 children with spastic CP participated for the reliability study. Their knee and/or ankle joints showed spasticity symptoms (catch or clonus) and did not have 1) severe deformities and 2) botulinum toxin within the last 4 months [42]. All guardians of the children gave written informed consents approved by the Pusan National University Yangsan Hospital IRB prior to the experiment (No.05–2015-117). Using the cMTS and iMTS, two experienced clinicians (a medical doctor and a physical therapist) examined the children, along with a volunteer who only performed the goniometric measurements for a blinded test in the cMTS. Please note that the volunteer was a clinical researcher trained to obtain goniometric measurements in clinical practice.

**Table 4:** Characteristics of study population

Characteristics	Group 1 (*n* = 18) for knee joint	Group 2 (*n* = 10) for ankle joint
Age (years)	7.5 ± 3.1	5.5 ± 3.5
Weight (kg)	25.1 ± 14.5	15.1 ± 8.3
Height (cm)	119.7 ± 21.1	101.8 ± 23.5
Male / Female	11 / 7	6 / 4
hemiplegia / bilateral / quadriplegia	5 / 9 / 4	2 / 5 / 3
GMFCS	I: 3, II: 8, III: 2, IV: 3, V: 2	I: 1, II: 1, III: 4, IV: 0, V: 4

GMFCS: gross motor function classification scale [1]

### Protocols

In the accuracy study, the subjects were placed in the supine position with markers placed, and two IMUs were attached with straps (Table 3). After several practices, the subjects performed the slow stretch motion as shown in Table 2 and maintained the posture at the end of the ROM for a few seconds to mimic the slow stretch of the cMTS to measure the ROM. Thereafter, the subjects performed the fast stretch motion. All subjects acted on their dominant legs and repeated the motions three times. Since it was difficult to capture the passive stretch without marker occlusion owing to the rater [41], we used the voluntary movements for this study. For better simulation of the cMTS, we asked the subjects to move the manipulated segment only and to perform the fast stretch within 1 s [10].

**Fig. 8:**
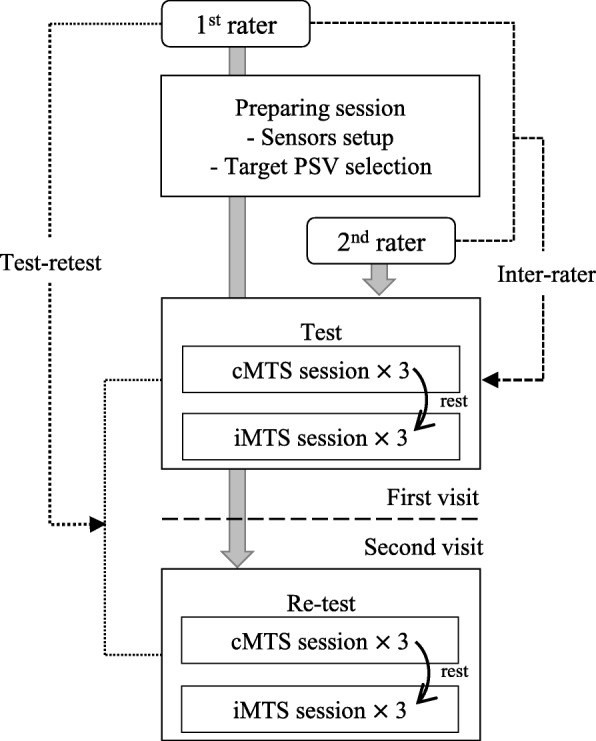
Protocol of the reliability study. The 1st rater performed the preparing session before the test, and the 2nd rater performed the actual test. The 1st rater performed a re-test within 3 days after the initial test

The protocol of the reliability study, which included the test-retest and inter-rater reliabilities of the cMTS and iMTS, is illustrated in Fig. 8. The first rater (clinician) attached the IMUs and the EMG sensors to the subjects and conducted several fast stretches to determine the target PSV for the iMTS (see the second subsection of method section on visual biofeedback). Then, all raters used the target PSV for visual biofeedback during fast stretches (Fig. 8). It should be noted that a fixed session order, conducting the iMTS sessions after finishing the cMTS sessions (Fig. 8), was required to prevent the rater from learning to target PSV prior to cMTS session.

For the cMTS session, the rater then performed the slow and the fast stretches to measure the ROM (R2) and AMR (R1), respectively, and each stretch was repeated three times [39]. Whenever the rater stopped at the end of the ROM or stopped/repositioned the subjects’ posture at the muscle reaction, the volunteer measured the joint angle of the posture using a goniometer based on the standard measurement method [6]. Note that we collected the data from the IMUs in this session to investigate how the goniometric measurement affects the reliability of the cMTS.

After the cMTS session, the participants were given an adequate rest period (more than 10 min) to minimize the effect of the fixed session order (Fig. 8). Next, the first rater performed the iMTS session using the visual biofeedback with the target PSV determined earlier. As in the cMTS, the iMTS session consisted of three slow and fast stretches (Fig. 8). If the rater failed to provide the target PSV despite the visual biofeedback, additional fast stretch trials were allowed to obtain three valid fast stretch trials with the target PSV [25]. Although the iMTS do not require the goniometric measurement, the volunteer additionally did the measurement in this session to determine the effect of PSV regulation due to the visual biofeedback of the iMTS.

Thereafter, the subjects rested for more than 15 min, and the second rater conducted the same cMTS and iMTS sessions with the subjects to evaluate the inter-rater reliability (Fig. 8). Note that both raters’ target PSV in the iMTS were identical. To evaluate the test-retest reliability, the first rater repeated the cMTS and iMTS sessions with the same subject within 3 days (Fig. 8).

### Data analysis

**Fig. 9:**
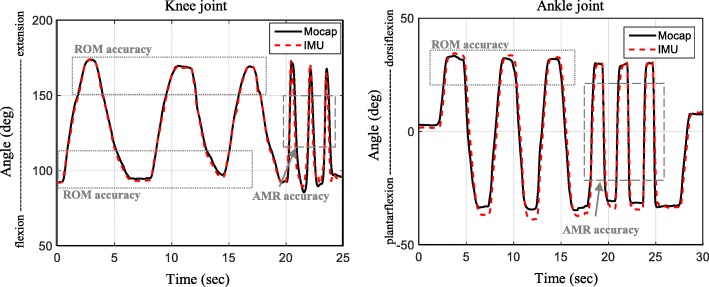
Representative joint angle comparison between the IMU and Mocap

In the accuracy study, we compared the joint angle estimated using the proposed algorithm in Fig. 3 with that measured by Mocap using the Nexus motion analysis software (Vicon, UK), as displayed in Fig. 9. In the MTS, the rater measured the ROM after stopping the slow stretch and recognized the AMR in the middle of the fast stretch. Hence, based on (2), we opted for the quasi-static period in the slow stretch motion to evaluate the ROM accuracy and the dynamic period in the fast stretch motion to evaluate the AMR accuracy (Fig. 9). To evaluate the accuracy quantitatively, the root mean square error (RMSE) between two joint angles during the periods were obtained using the MATLAB. Of the quasi-static periods, the periods that correspond to the ankle dorsi-flexor were excluded from the calculation of the RMSE (Fig. 9b). We used two-way ANOVA to test for the difference in accuracy of the proposed algorithm between the motions (knee extension/flexion and ankle dorsiflexion) that corresponded to the target muscles as well as between the outcomes (ROM and AMR).

According to the MTS assessment procedure shown in Fig. 1, the test-retest and inter-rater reliabilities were analyzed using the ROM (R2), AMR (R1), and spasticity angle (SA; difference between R2 and R1) obtained from the cMTS and iMTS sessions. The reliability was quantitatively evaluated by intraclass correlation coefficients (ICC), which were calculated using the SPSS software version 23 (IBM corporation., USA). Note that ICC > 0.8 indicates good consistency; ICC > 0.6 moderate consistency; and below (ICC < 0.6) poor consistency [43, 44]. Moreover, to investigate the cause of reliability deterioration of the cMTS, we additionally calculated the ICCs of the AMR obtained from 1) the IMU (calculating the joint angle and detecting the muscle reaction) in the cMTS sessions and from 2) the goniometer in the iMTS sessions. The former, cMTS with IMU, was used to show the effect of goniometric measurements and the latter, cMTS with visual biofeedback, to determine the effect of unregulated PSVs.

The duration of clonus obtained by the IMU was compared with that measured by the EMG. The RMSE and ICC between the two were obtained for evaluation. It is noteworthy that the method via EMG in determining the duration was the same as that (9) via IMU [39].

In addition, we attempted to confirm the effectiveness of the visual biofeedback. It was evaluated by the achieving rate of the target PSV as follows [25]:

10 $$ {\uplambda}_{\mathrm{ar}}\left(\%\right)=\frac{3}{{\mathrm{n}}_{\mathrm{total}}}\times 100 $$

where n_total_ denotes the required number of stretches to achieve the target PSV three times.

## Results

### Accuracy study

The RMSE of the joint angles showing the ROM accuracy (quasi-static periods in slow stretch motions) and AMR accuracy (dynamic periods in fast stretch motions) is summarized in Table 5. The RMSEs were less than 4° in all cases. For all motions, the RMSE for the AMR was larger than the RMSE for the ROM, and those RMSEs were statistically different (*p* = 0.006), showing the difference between the accuracies of ROM and AMR. Compared to knee extension, knee flexion and ankle dorsiflexion resulted in larger RMSE. The difference in the RMSEs between the motions was statistically significant (*p* = 0.045).

**Table 5:** RMSE between the Mocap and IMU

Motion		ROM accuracy (deg)		AMR accuracy (deg)	
		Mean (SD)	RMSE (SD)	Mean (SD)	RMSE (SD)
Knee extension	Mocap	165.05 (6.48)	2.24 (1.55)	145.68 (7.31)	2.95 (1.07)
	IMU	164.16 (7.20)		147.53 (7.47)	
Knee flexion	Mocap	72.92 (5.43)	3.05 (1.84)	104.21 (8.34)	3.97 (2.02)
	IMU	74.58 (6.91)		105.53 (9.78)	
Ankle dorsiflexion	Mocap	32.92 (5.11)	3.11 (2.91)	5.91 (6.61)	3.86 (1.86)
	IMU	30.08 (3.03)		7.62 (7.65)	

SD denotes standard deviation

### Reliability study

#### Test-retest and inter-rater reliabilities

The test-retest reliabilities of the cMTS and iMTS are summarized in Table 6. The cMTS showed a poor (AMR) to moderate (ROM and SA) consistency for the knee flexor, poor (AMR and SA) to moderate (ROM) consistency for the knee extensor, and moderate (AMR and SA) to good (ROM) consistency for the ankle plantar-flexor; the iMTS showed good consistency in all cases.

**Table 6:** Test-retest and inter-rater reliabilities

			Test-retest reliability			Inter-rater reliability		
			Test (deg) Mean (SD)	Re-test (deg) Mean (SD)	ICC (95% CI)	1st Rater (deg) Mean (SD)	2nd Rater (deg) Mean (SD)	ICC (95% CI)
Knee flexor	ROM (R2)	cMTS	139.19 (10.48)	140.01 (8.59)	0.71 (0.38–0.80)	139.19 (10.48)	139.66 (8.35)	0.65 (0.43–0.77)
		iMTS	144.73 (11.61)	142.34 (8.94)	0.84 (0.55–0.92)	144.73 (11.61)	142.34 (8.94)	0.81 (0.65–0.89)
	AMR (R1)	cMTS	122.94 (15.74)	120.27 (12.60)	0.50 (0.31–0.70)	122.94 (15.74)	125.88 (15.05)	0.50 (0.17–0.73)
		iMTS	126.59 (14.82)	126.06 (14.32)	0.89 (0.80–0.95)	126.59 (14.82)	126.78 (14.75)	0.86 (0.63–0.95)
	SA (R2-R1)	cMTS	16.25 (12.38)	19.74 (11.09)	0.63 (0.27–0.78)	16.25 (12.38)	19.74 (11.09)	0.41 (0.48–0.61)
		iMTS	18.63 (10.30)	17.76 (10.01)	0.81(0.74–0.91)	18.63 (10.30)	17.47 (10.56)	0.80 (0.53–0.90)
Knee extensor	ROM (R2)	cMTS	37.43 (16.16)	46.00 (14.82)	0.73 (0.35–0.79)	37.43 (16.16)	45.01 (19.02)	0.79 (0.50–0.85)
		iMTS	47.91 (15.61)	46.94 (9.86)	0.83 (0.73–0.93)	47.91 (15.61)	45.25 (17.28)	0.88 (0.57–0.92)
	AMR (R1)	cMTS	39.49 (18.38)	45.76 (14.82)	0.64 (0.32–0.78)	39.49 (18.38)	54.68 (17.82)	0.59 (0.46–0.84)
		iMTS	49.97 (16.08)	47.73 (8.92)	0.86 (0.74–0.93)	49.97 (16.08)	58.59 (16.01)	0.84 (0.70–0.92)
	SA (R2-R1)	cMTS	−4.48 (3.59)	−5.15 (4.92)	0.40 (0.24–0.59)	− 4.48 (3.59)	−9.67 (5.38)	0.50 (0.10–0.73)
		iMTS	−5.20 (5.43)	−4.96 (4.46)	0.81 (0.63–0.91)	−5.20 (5.43)	−12.13 (9.47)	0.80 (0.54–0.86)
Ankle plantar-flexor	ROM (R2)	cMTS	22.77 (7.49)	24.44 (9.50)	0.94 (0.43–0.97)	22.77 (7.49)	21.21 (7.82)	0.94 (0.76–0.91)
		iMTS	22.29 (10.57)	21.59 (8.78)	0.92 (0.65–0.89)	22.29 (10.57)	24.78 (8.86)	0.92 (0.66–0.92)
	AMR (R1)	cMTS	3.33 (7.57)	4.67 (6.12)	0.71 (0.46–0.78)	3.33 (7.57)	8.24 (9.82)	0.63 (0.38–0.83)
		iMTS	8.27 (3.29)	6.40 (4.88)	0.83 (0.55–0.90)	8.27 (3.29)	10.27 (6.92)	0.83 (0.61–0.91)
	SA (R2-R1)	cMTS	19.44 (7.72)	19.47 (2.63)	0.63 (0.01–0.45)	19.44 (7.72)	12.97 (9.92)	0.19 (0.04–0.59)
		iMTS	14.02 (8.44)	15.19 (9.87)	0.80 (0.68–0.96)	14.02 (8.44)	14.51 (9.46)	0.81 (0.68–0.89)

CI denotes the confidence interval

Table 6 shows the results of the inter-rater reliability. The proposed iMTS showed good consistency in all the muscles (joints), whereas the cMTS showed good consistency in the ROM of the ankle plantar-flexor only. The AMR of the knee flexor and all SA of the cMTS were poor, and the other AMR (knee extensor and ankle plantar-flexor) and ROM (knee flexor and extensor) of the cMTS showed moderate consistency.

#### Causes of reliability deterioration in the MTS assessment

The comparison on the reliabilities (ICC) of the AMR under the cMTS, iMTS, and two modified cMTS by adding parts of the iMTS is summarized in Table 7. In all muscles, both the modified cMTS had better test-retest and inter-rater reliabilities than the cMTS, and had worse reliabilities than the proposed iMTS. However, it was not clear which modification results in a more significant improvement of the reliability.

**Table 7:** Cause of reliability deterioration of AMR in the MTS assessment

Test-retest (ICC)	Knee flexor	Knee extensor	Ankle plantar-flexor
cMTS	0.50 (0.31–0.70)	0.64 (0.32–0.78)	0.71 (0.46–0.78)
cMTS with IMU	0.78 (0.51–0.88)	0.74 (0.44–0.90)	0.77 (0.54–0.87)
cMTS with VB	0.71 (0.49–0.79)	0.82 (0.60–0.91)	0.79 (0.67–0.86)
iMTS	0.89 (0.80–0.95)	0.86 (0.74–0.93)	0.83 (0.55–0.90)
Inter-rater (ICC)	Knee flexor	Knee extensor	Ankle plantar-flexor
cMTS	0.50 (0.17–0.73)	0.59 (0.46–0.84)	0.63 (0.38–0.83)
cMTS with IMU	0.65 (0.49–0.76)	0.78 (0.67–0.84)	0.69 (0.59–0.77)
cMTS with VB	0.76 (0.53–0.81)	0.75 (0.66–0.82)	0.72 (0.61–0.82)
iMTS	0.86 (0.63–0.95)	0.84 (0.70–0.92)	0.83 (0.61–0.91)

All data are presented as ICCs (95% CI); VB denotes visual biofeedback

#### Duration of clonus

Table 8 shows the agreement of the duration of clonus between the iMTS and conventional EMG methods. On average, there was a small error (~ 0.07 s), and the high ICC showed good consistency between both durations.

**Table 8:** Duration of clonus

	Mean (SD)	ICC (95% CI)
iMTS	7.02 (5.01)	0.96 (0.89–0.98)
EMG	7.09 (4.78)	

#### Achieving rate of the target PSV

The calculated achieving rates using (10) are summarized in Table 9. The mean achieving rate was ~ 77%, which indicated that approximately four stretches have been performed to provide three successful PSVs. Note that the second rater participated only in the test trials (Fig. 8).

**Table 9:** Achieving rate of the target PSV

	1st rater		2nd rater
	Test (%)	Re-test (%)	Test (%)
Knee flexor	75	80	78
Knee extensor	77	75	83
Ankle plantar-flexor	80	76	77
Average (%)	77		

## Discussion

This study was conducted to overcome the limitations of the MTS in terms of its accuracy and reliability by proposing the iMTS. To improve its accuracy, we proposed an IMU-based joint angle calculation algorithm as a part of the iMTS. Despite a magnetometer not being used in the IMU, the proposed algorithm reduced the joint angle measurement error of the cMTS (less than 10° [45]) by about 69% (about 3° RMSE in Table 5) on the knee joint. The accuracy of the proposed algorithm was comparable to the existing algorithm with magnetometer [21–23], (about 4° RMSE) and without magnetometer (from 5° to 8° RMSE) [3, 12].

The significant difference in the RMSE between the ROM and AMR shows that the AMR error was larger than the ROM error. It was because AMR measurement, which was conducted by (4) in the dynamic state, would be vulnerable to drift errors of the gyroscope in the IMU. However, owing to the short dynamic period regarding the AMR (< 1 s) in the MTS assessment, the drift error due to the integration in (4) did not occur significantly (Table 5). The accuracy difference according to the motions would result from the anatomical characteristics of the joints; the non-sagittal plane movement of the ankle (inversion) was larger than that of the knee (internal/external rotation), as mentioned in the first subsection of method section. Since the proposed algorithm used a mapping scheme using rotation matrix to compensate for the movements, as shown in (6), (7), and (8), the RMSEs of the ankle motion were still relatively small (Table 5).

In addition to the joint angle calculation algorithm, the proposed iMTS contains a muscle reaction detection method as well as a visual biofeedback mechanism to improve the reliability by considering the velocity-dependent characteristics of muscle reaction. Our clinical tests showed that the test-retest and inter-rater reliabilities of the proposed iMTS significantly improved compared with those of the cMTS. Moreover, the reliability of the iMTS (good consistency in all cases) was better in this study than in the existing studies on improving the MTS in the lower extremities [22]. The SA, which was determined by the AMR and ROM, showed slightly lower reliability than the AMR (Table 6). It was because the iMTS still used the rater’s subjective haptic feeling to decide the end of the ROM. In fact, the ROM had a lower reliability than the AMR (Table 6), while the ICCs of the ROM in the iMTS were higher than the reported ICCs of the conventional ROM measurement (< 0.79) [43].

From the comparison study, we found that the deterioration in reliability of the MTS assessment is due to the combined causes of goniometric measurement and unregulated PSV. It can be supported that all modified conditions of the cMTS (cMTS with IMU and cMTS with VB) still showed poor to moderate consistency (Table 7). The fact that there was no dominant cause between the two shows why the iMTS was proposed by integrating the joint angle calculation algorithm with abnormal muscle reaction detection and visual biofeedback.

The main outcome of the MTS was the SA, the difference between ROM and AMR, which distinguishes the neural (dynamic spastic) component from total resistance (hyper-resistance) during passive stretching [2, 46, 47]. Since it is well-known that the SA is closely related to the therapeutic effect of botulinum toxin type A injection (BTX-A) [2, 48], the SA measurement was consistent because iMTS contributes to 1) successful rehabilitation management (e.g., BTX-A without surgery) [49] and 2) better efficacy and safety of BTX-A through dose adjustment based on reliable SA measurement [50]. Moreover, the reliable ROM/AMR measurement of the iMTS is beneficial to physical/occupational therapy and orthotic treatment [51].

In addition to ROM, AMR, and SA, the proposed iMTS can also evaluate another key part of MTS, clonus, while clonus was not considered in existing studies on improving the MTS assessment [3, 21, 22]. For clonus, the ankle was included to the target joint, and the IMU-based muscle reaction detection method was developed to detect the clonus accurately with the catch. We also added the function to measure the duration of clonus to distinguish between fatigable and unfatigable clonus. The accuracy of the measurement was comparable to that of the measurement via EMG (Table 8).

Compared with existing studies, the present study attempted to develop an improved MTS assessment system that is practical enough for clinical use. The proposed iMTS followed the procedure of the cMTS, and the sensor attachment location was determined by considering the anatomical characteristics (Table 3). Moreover, the iMTS contains a novel IMU-based joint angle calculation without magnetometer, considering the clinical setting; the magnetometer for joint angle calculation requires inconvenient calibration procedure due to local distortion of the earth’s magnetic field caused by unknown materials and magnetic objects in outpatients and medical devices, such as ultrasound and transcutaneous electrical nerve stimulation [20]. We also developed a visual biofeedback to provide regular PSVs for clinicians’ convenient use [25]; thus, the high success rate of providing target PSVs was obtained (Table 9).

This study has several limitations. The iMTS assumed that the rater can check whether muscle reaction exists (#1 in Fig. 1) and whether it was clonus (#3 in Fig. 1). Although this is not a strong assumption, especially when the rater is a well-experienced clinician, the iMTS needs to determine them autonomously, including the spasticity grade [2, 11] for a more objective spasticity assessment. For clinical use, the target PSV need to fixed to compare the level of spasticity between subjects. Since there is no clear standard to determine PSV for MTS assessment, especially iMTS, standardization of the target PSV may be required. The experimental verification of the accuracy of iMTS was conducted for healthy subjects although there may be a difference in the movements between healthy subjects and patients. Even though the proposed joint angle calculation algorithm can compensate some non-sagittal movements (knee internal/external rotation and ankle inversion/eversion) that are noticeable even in healthy people due to the anatomical characteristics of knee and ankle joints, some bony deformities of CP children (e.g., foot equinus and hindfoot valgus [52]) can cause non-sagittal movements that the algorithm cannot compensate for. Hence, the low accuracy due to those deformities needs to be improved in future work. In addition, we need to expand the target muscles by including the ankle dorsi-flexor that was excluded owing to its relatively lower clinical importance and the upper limb muscles, such as the elbow flexor [23] for stroke patients.

## Conclusions

In this paper, we proposed a novel sensor-based spasticity assessment system to improve the accuracy and reliability of a well-known clinical instrument for spasticity, namely, MTS. For the IMU-based MTS assessment, with consideration to the clinical environment, we developed a magnetometer-free joint angle calculation method, a muscle reaction (catch and clonus) detection function, and a visual biofeedback method to help regulate PSV. The accuracy of the proposed system was validated through a comparison with a motion capture system, and the reliability of the system was evaluated by conducting a clinical spasticity assessment of the lower limbs (knee and ankle joints) of 28 children with cerebral palsy. With high accuracy of the joint angle calculation (RMSE < 3 deg), the results of the clinical test showed that the proposed system can significantly improve test-retest and inter-rater reliabilities of MTS (good consistency; ICC > 0.8) compared to conventional MTS (poor or moderate consistency; 0.2–0.6 ICC). With the proposed system, it was also noted that the deterioration in reliability of conventional MTS assessment is due to the combination of goniometric measurement and unregulated PSV.

## Abbreviations

AMR: Angle of muscle reaction
AOC: Angle of catch
BTX-A: Botulinum toxin type A injection
cMTS: Conventional modified Tardieu scale assessment
CP: Cerebral palsy
GUI: Graphic user interface
IAOC: Initial angle of clonus
ICC: Intraclass correlation coefficients
iMTS: IMU-based modified Tardieu scale assessment
IMU: Inertial measurement unit
IRB: Institutional review boards
MAS: Modified Ashworth scale
Mocap: Motion capture system
MTS: Modified Tardieu scale
PSV: Passive stretch velocity
RMSE: Root mean square error
ROM: Range of motion
SA: Spasticity angle
